# Long-term safety and efficacy of renal sympathetic denervation in atrial fibrillation: 3-year results of the AFFORD study

**DOI:** 10.1007/s00392-023-02222-3

**Published:** 2023-05-25

**Authors:** Victor J. M. Zeijen, Dominic A. Theuns, Lida Feyz, Kari A. Saville, Rohit Bhagwandien, Isabella Kardys, Nicolas M. Van Mieghem, Joost Daemen

**Affiliations:** Department of Cardiology, Thorax Center, Erasmus University Medical Center, Room Rg-628, P.O. Box 2040, 3000 CA Rotterdam, The Netherlands

**Keywords:** Atrial fibrillation, Anti-arrhythmia agents, Electrocardiography, Ambulatory, Hypertension, Kidney, Sympathectomy

## Abstract

**Background:**

Atrial fibrillation (AF) is the most common sustained arrhythmia which has been associated with increased sympathetic nervous system activity and hypertension. Recent evidence indicated that renal sympathetic denervation (RDN) could safely contribute to an improvement in AF burden.

**Objective:**

To investigate the long-term safety and efficacy of radiofrequency RDN in hypertensive patients with symptomatic AF.

**Methods:**

This pilot study included patients with symptomatic paroxysmal or persistent AF (European Hearth Rhythm Association class ≥ II) despite optimal medical therapy, office systolic blood pressure (BP) ≥ 140 mmHg and ≥ 2 antihypertensive drugs. AF burden was measured using an implantable cardiac monitor (ICM), implanted 3 months prior to RDN. ICM interrogation and 24-h ambulatory BP monitoring were performed at baseline and at 3/6/12/24/36 months post RDN. The primary efficacy outcome was daily AF burden. Statistical analyses were performed using Poisson and negative binomial models.

**Results:**

A total of 20 patients with a median age [25th–75th percentiles] of 66.2 [61.2–70.8] years (55% female) were included. At baseline, office BP ± standard deviation (SD) was 153.8/87.5 ± 15.2/10.4 mmHg, while mean 24-h ambulatory BP was 129.5/77.3 ± 15.5/9.3 mmHg. Baseline daily AF burden was 1.4 [0.0–10.9] minutes/day and throughout a 3-year follow-up period, no significant change was observed (− 15.4%/year; 95% confidence interval (CI) − 50.2%, + 43.7%; *p* = 0.54). The number of defined daily doses of antiarrhythmic drugs and antihypertensive drugs remained stable over time, while mean 24-h ambulatory systolic BP decreased with − 2.2 (95% CI − 3.9, − 0.6; *p* = 0.01) mmHg/year.

**Conclusions:**

In patients with hypertension and symptomatic AF, stand-alone RDN reduced BP but did not significantly reduce AF burden up until 3 years of follow-up.

**Graphical abstract:**

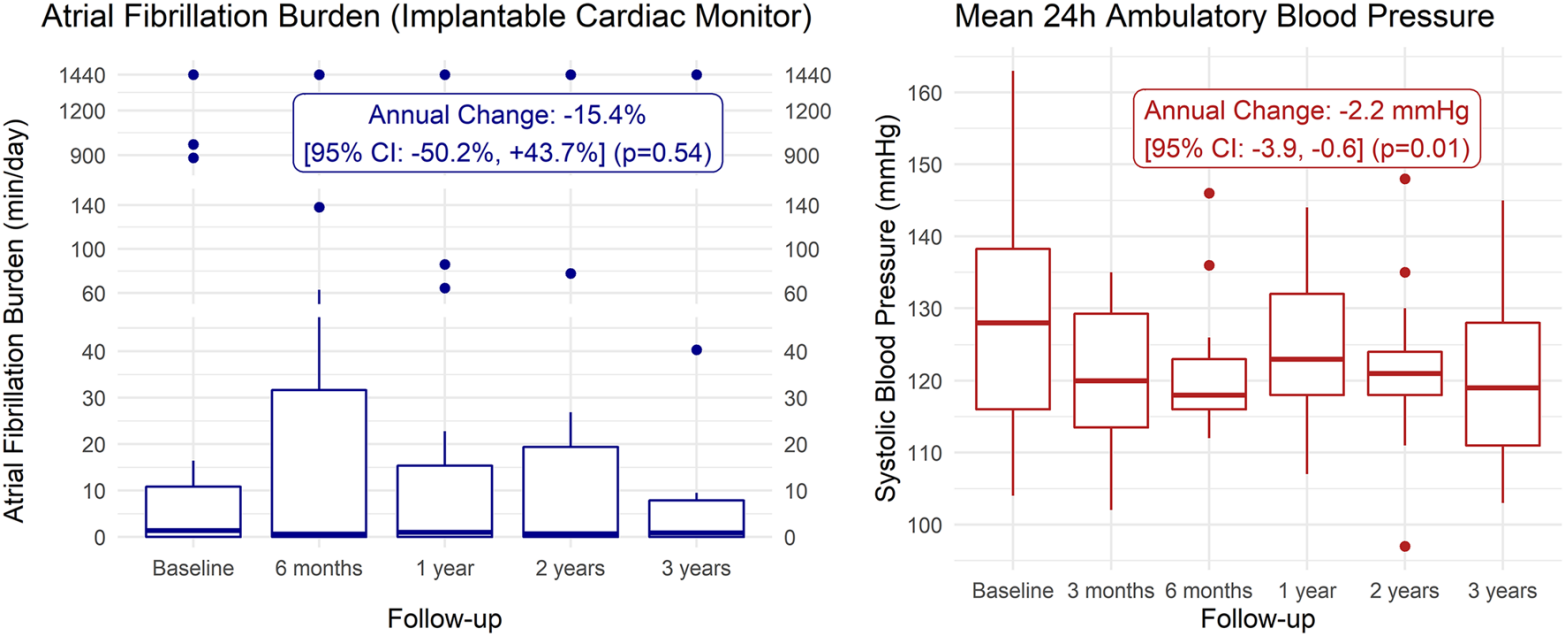

**Supplementary Information:**

The online version contains supplementary material available at 10.1007/s00392-023-02222-3.

## Introduction

Atrial fibrillation (AF) is an increasingly common cardiac arrhythmia currently affecting 33.5 million patients worldwide [1]. As AF increases the risk of stroke, dementia, heart failure, myocardial infarction and all-cause mortality, this condition accounts for nearly six million disability-adjusted life-years (DALYs) worldwide [2, 3]. Hypertension has been recognized as one of the most important risk factors for the development of AF [4].

Guideline-recommended treatment of AF involves symptom control (either rate or rhythm control), stroke prevention and cardiovascular risk management [5]. Whereas symptom control using pharmacotherapy is recommended in newly diagnosed patients, catheter ablation (i.e., pulmonary vein isolation; PVI) should be considered in those who remain symptomatic despite optimal medical therapy [5]. Nevertheless, current treatment strategies remain suboptimal as 61.8% of all patients with AF remain symptomatic (European Hearth Rhythm Association (EHRA) class ≥ II) and up to 16.5% may suffer from severe or disabling symptoms (EHRA class III or IV) [6].

Renal sympathetic denervation (RDN) has emerged as a minimally invasive treatment modality for diseases associated with sympathetic overactivity. To date, multiple sham-controlled randomized clinical trials demonstrated the significant BP-lowering effect of RDN in the absence of major safety concerns [7–13]. In hypertensive patients with symptomatic AF, RDN in addition to PVI proved to lower AF recurrence rate at 1 year by 38% as compared to PVI alone [14]. In parallel, RDN demonstrated to lower the risk of new-onset or recurrent AF in hypertensive patients with sinus rhythm and a high risk of developing AF [15].

The aim of the current pilot study was to evaluate the long-term safety and efficacy of RDN as a stand-alone treatment modality for patients with symptomatic AF and hypertension.

## Methods

### Study design and population

The current pilot study was a prespecified 3-year follow-up analysis of all patients enrolled in the single-center, single-arm AFFORD study (*n* = 20). Details on the study design and entry criteria have been published previously [16]. In short, patients with symptomatic paroxysmal or persistent AF (EHRA class ≥ II), a history of hypertension (office systolic BP ≥ 140 mmHg while prescribed ≥ 2 antihypertensive drugs) and an estimated Glomerular Filtration Rate (eGFR) ≥ 45 ml/min/1.73 m^2^ were included. This study was preregistered in the Dutch trial registry (clinicaltrialregister.nl, NTR number: NTR5329) and was approved by the local ethics committee. The study was conducted in accordance with the Declaration of Helsinki and all patients provided written informed consent.

### Screening and procedure

After the baseline visit, 3 months prior to the RDN procedure, eligible patients were scheduled for implantation of a SJM Confirm DM2102 implantable cardiac monitor (ICM) (St. Jude Medical, St. Paul, Minneapolis, United States of America). After 3 months, ICM interrogation was performed to determine baseline AF burden. All patients subsequently underwent radiofrequency RDN using the St. Jude EnligHTN™ system (St. Jude Medical, St. Paul, Minneapolis, United States of America) [16].

### Outcomes

The primary efficacy outcome was the temporal evolution of AF burden (in minutes/day) as based on ICM interrogation up to 3 years following RDN. The primary outcome was assessed separately in (I) the entire cohort and (II) in a subgroup of patients who did not develop any permanent or long-standing persistent AF throughout the course of the study. Secondary efficacy outcomes were the number of AF episodes, the cumulative duration of AF (in minutes) and the maximal ventricular rate response (VRR) as based on ICM interrogation, heart rate and the number of supraventricular and ventricular ectopic beats as based on 24-h Holter monitoring, EHRA class, quality-of-life, ambulatory BP (mean 24 h, daytime, nighttime), office BP and the number of defined daily dosages (DDD) and classes of antiarrhythmic and antihypertensive drugs [17].

The primary safety outcome was a composite endpoint consisting of electrical or chemical cardioversion, PVI, MAZE procedure, uptitration of antiarrhythmic drugs as compared to baseline or increase in EHRA class as compared to baseline. Secondary safety outcomes were all individual components of the composite endpoint.

### Follow-up data collection

Scheduled outpatient clinic visits were performed at baseline (3 months prior to RDN), 1, 3 and 6 months and yearly up to 3 years following RDN.

ICM interrogation was performed under supervision of an experienced certified cardiac device specialist (DT) before RDN and at 6 months and 1, 2, 3 years post RDN. Up until 1-year follow-up, all saved episodes of arrhythmias within 3 months before interrogation were assessed. Afterwards, episodes of arrhythmias within 1 year before interrogation were assessed and adjusted to an equal time window (i.e., 3 months). All stored episodes of arrhythmias were manually classified by two authors (VZ and DT). When ICM interrogation could not be performed in the desired timeframe for technical reasons (e.g., battery end-of-life), the latest available readings were analyzed and adjusted to a 3-month time window. In patients with permanent AF who experienced a lack of ICM storage space, AF burden was imputed to 24 h/day to prevent underestimation of the daily AF burden. In addition, a sensitivity analysis excluding patients with permanent or long-standing persistent AF was performed.

**Fig. 1:**
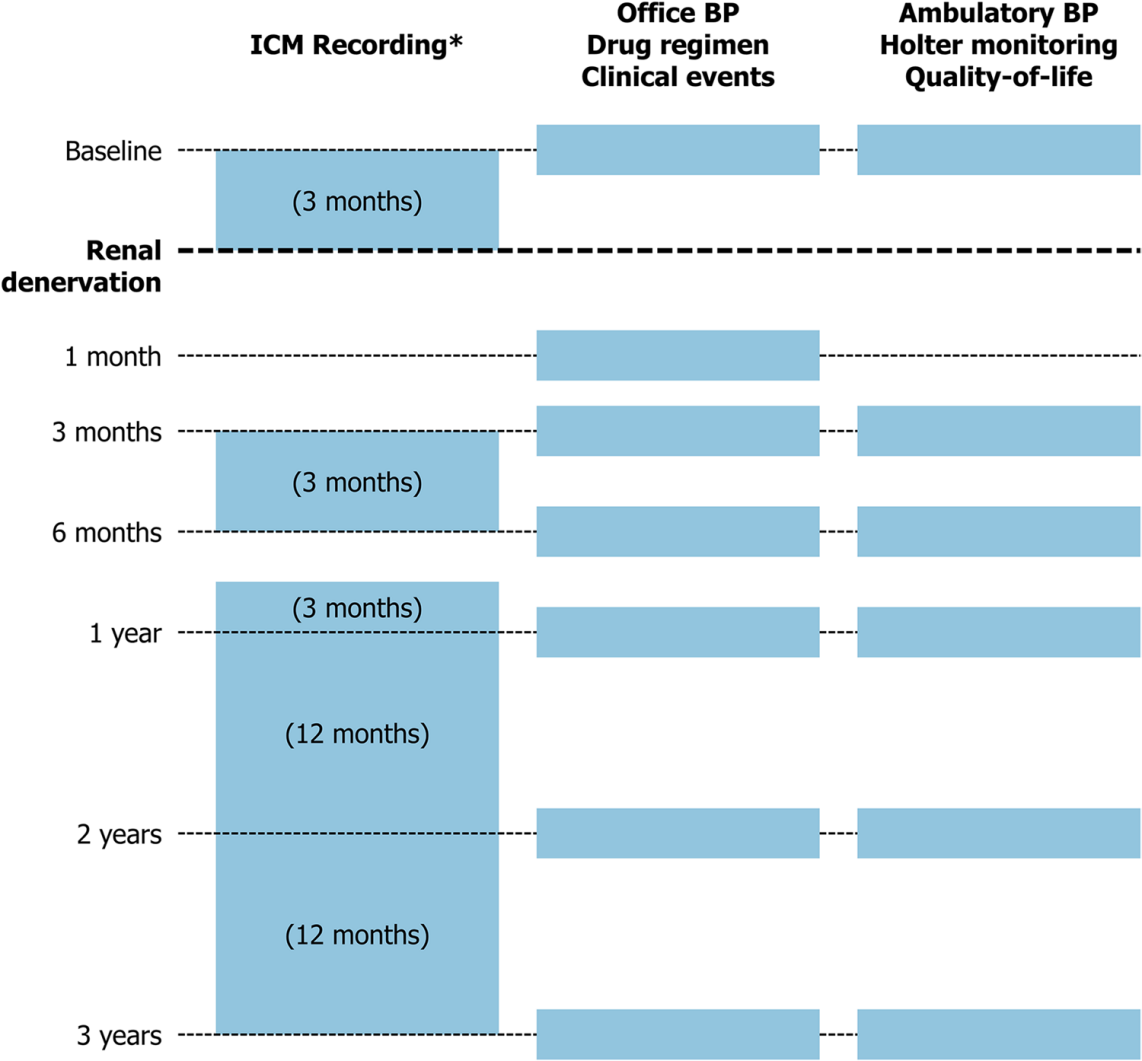
Study assessments flowchart. *All AF burden measures were adjusted to a 3-month time window to allow for comparability over time. *BP* blood pressure. *ICM* implantable cardiac monitor

Ambulatory BP monitoring was performed using the Spacelabs 90217A monitor device (Spacelabs Healthcare, Snoqualmie, Washington, United States of America) at baseline, 3, 6 months and 1, 2, 3 years. Office BP measurement was performed at all visits using the Omron M7 Intelli IT device (OMRON Healthcare Europe, Hoofddorp, The Netherlands). Holter monitoring was performed at baseline, 3 and 6 months and 1, 2, 3 years using the Evo Digital Recorder device (Spacelabs Healthcare, Snoqualmie, Washington, United States of America). Quality-of-life was assessed at baseline, 3, 6 months and 1, 2, 3 years using the dedicated Atrial Fibrillation Effect on QualiTy-of-Life (AFEQT) questionnaire including the overall AFEQT score (range 0–100) [18]. Data on drug regimen and safety outcomes were collected at all study visits (Fig. 1).

### Statistical analysis

Continuous variables were reported as mean ± standard deviation (SD) or median [25th–75th percentiles] for normally and non-normally distributed variables, respectively. Normality was assessed using histograms and quantile–quantile plots. Categorical variables were reported as number of patients (percentage).

The primary efficacy outcome (AF burden in minutes/day) and several secondary efficacy outcomes (i.e., number and total duration of AF burden, supraventricular and ventricular ectopic beats and DDDs and number of antiarrhythmic and antihypertensive drugs) were considered count data and were consequently analyzed using either Poisson or negative binomial regression. Poisson regression was applied in case the variance was smaller than or equal to the mean of the outcome variable, whereas negative binomial regression was applied when the variance was greater than the mean. Continuous variables (i.e., heart rate, maximal VRR, ambulatory and office blood pressure, overall AFEQT score) were analyzed using linear mixed-effects models. Ordinal categorical variables (i.e., EHRA class and AFEQT patient’s last awareness of AF) were analyzed using cumulative link mixed models. Individual classes of antiarrhythmic drugs were displayed as patients (percentages) only and no formal statistical testing was performed.

All models were fitted with the outcome variable as dependent variable while the covariate follow-up time was included in the fixed-effects part and random intercepts on the patient-level were fitted to account for repeated measurements of the outcome over time. Regression coefficients [including 95% confidence intervals (CI) and *p *values] for the effect of follow-up time were reported. For Poisson/negative binomial models, the percentages change per year in outcome (calculated as the exponent of the regression coefficient minus one afterwards multiplied by 100%) were reported, whereas for linear mixed-effect models, the crude regression coefficient was reported, reflecting the numerical change in the outcome variable (in original units) per year. For cumulative link mixed models, only *p *values were reported.

For the primary and secondary safety outcomes, patients were censored at the 3-year follow-up visit, at loss-to-follow-up or when the event-of-interest occurred (whichever occurred first). Cumulative incidence estimates and the median time-to-event (including 25th–75th percentiles) were derived from the Kaplan–Meier function.

This study was a pilot study which was therefore not statistically powered to detect a predetermined size of effect for any of the outcomes. All statistical tests were two-tailed and *p* values < 0.05 were considered statistically significant. Analyses were performed using R version 4.1.1 with the packages “nlme”, “lme4”, “GLMMadaptive”, “ordinal”, “survival” and “ggplot2” [19].

## Results

### Study population

For the current long-term follow-up study, 20 patients were included. All patients completed 2-year follow-up and 19 (95%) completed 3-year follow-up, resulting in a total number of 98 observations. Successful ICM interrogation could not be performed for 6 observations due to technical reasons (i.e., battery end-of-life or device error), while ICM interrogation was performed early (as compared to the study protocol) for 4 observations. Median age at baseline was 66.2 [61.2–70.8] years and 11 (55%) patients were female. Office BP at baseline was 153.8/87.5 ± 15.2/10.4 mmHg and mean 24-h ambulatory BP was 129.5/77.3 ± 15.5/9.3 mmHg while patients were prescribed 2.7 ± 1.6 DDDs of antihypertensive drugs. Eighteen patients (90%) had a history of paroxysmal AF, whereas two patients (10%) had persistent AF. Daily AF burden at baseline was 1.4 [0.0–10.9] minutes/day while patients were prescribed a median of 1.0 [0.5–1.3] DDDs of antiarrhythmic drugs (Table 1).

**Table 1:** Baseline characteristics

Variable	Study population (*n* = 20)
Age (years), median [25th–75th percentiles]	66.2 [61.2–70.8]
Female sex, *n* (%)	11 (55)
Body Mass Index (kg/m^2^), mean ± SD	30.9 ± 5.6
Cardiovascular risk factors	
Hypertension, *n* (%)	20 (100)
Diabetes, *n* (%)	2 (10)
Dyslipidemia, *n* (%)	8 (40)
Smoking	
Ever-smoking, *n* (%)	4 (20)
Current smoking, *n* (%)	1 (5)
Family history of ischemic heart disease, *n* (%)	4 (20)
Arrhythmia-related history	
Atrial fibrillation	
Paroxysmal atrial fibrillation, *n* (%)	18 (90)
Persistent atrial fibrillation, *n* (%)	2 (10)
Cardioversion	
Electrical cardioversion, *n* (%)	10 (50)
Chemical cardioversion, *n* (%)	2 (10)
Flutter ablation, *n* (%)	1 (5)
Pulmonic vein isolation, *n* (%)	4 (20)
MAZE procedure, *n* (%)	0 (0)
Left Atrial Appendage Occluder, *n* (%)	2 (10)
Prescribed medication	
Antiarrhythmic drugs	
Defined daily dosages, median [25th–75th percentiles]	1.0 [0.5–1.3]
Number of drugs, median [25th–75th percentiles]	1.0 [1.0–1.3]
Antihypertensive drugs	
Defined daily dosages, mean ± SD	2.7 ± 1.6
Number of drugs, median [25th–75th percentile]	2.0 [2.0–3.0]
Blood pressure	
Mean 24-h ambulatory BP (mmHg), mean ± SD	129.5/77.3 ± 15.5/9.3
Daytime ambulatory BP (mmHg), mean ± SD	132.3/79.5 ± 15.8/9.7
Nighttime ambulatory BP (mmHg), mean ± SD	120.7/70.1 ± 17.4/10.4
Office BP (mmHg), mean ± SD	153.8/87.5 ± 15.2/10.4
Quality-of-life	
Overall AFEQT score (range 0–100), mean ± SD	66.4 ± 15.0

*AFEQT* Atrial Fibrillation Effect on QualiTy-of-Life, *BP* blood pressure, *EHRA* European Heart Rhythm Association, *SD* standard deviation

### Efficacy outcomes

Following RDN, daily AF burden as measured using an ICM did not significantly change throughout the 3-year time period (− 15.4%/year; 95% CI − 50.2%, + 43.7%; *p* = 0.54). The maximal VRR during AF increased throughout follow-up (+ 10.6 beats/minute/year; 95% CI + 2.8, + 18.4; *p* = 0.009). Similar results were observed in patients who did not convert to permanent AF during follow-up (*n* = 17) (Table 2).

**Table 2:** Atrial fibrillation burden as measured using an implantable cardiac monitor

All observations^a^	Baseline	6 months	1 year	2 years	3 years	Annual change (95% CI)	*p *value
	(*N* = 20)	(*N* = 19)	(*N* = 20)	(*N* = 20)	(*N* = 19)		
Daily AF burden (min/day), median [25th–75th percentiles]	1.4 [0.0–10.9]	0.7 [0.0–31.6]	1.0 [0.0–15.4]	0.7 [0.0–19.4]	0.9 [0.0–7.9]	− 15.4% (− 50.2%, 43.7%)	0.54
AF episodes (min), median [25th–75th percentiles]	125.0 [1.5–978.0]	44.0 [0.0–2833.0]	91.5 [0.0–1403.5]	63.9 [0.0–1769.4]	81.9 [0.4–718.6]	− 14.7% (− 56.2%, 66.3%)	0.64
Maximal ventricular rate response (beats/minute), mean ± SD	123.3 ± 23.4	120.7 ± 33.8	102.1 ± 24.4	145.5 ± 30.0	145.5 ± 37.7	10.6 (2.8, 18.4) beats/minute	0.009

Patients without any permanent AF^b^	Baseline	6 months	1 year	2 years	3 years	Annual change (95% CI)	*p *value
	(*N* = 17)	(*N* = 16)	(*N* = 17)	(*N* = 17)	(*N* = 16)		
Daily AF burden (min/day), median [25th–75th percentiles]	1.3 [0.0–6.9]	0.7 [0.0–29.5]	0.9 [0.0–3.3]	0.6 [0.0–5.5]	0.9 [0.2–6.3]	− 21.4% (− 54.7%, 36.6%)	0.39
AF episodes (*n*), median [25th–75th percentiles]	1.0 [0.8–2.5]	1.0 [0.0–7.8]	2.0 [0.0–13.0]	1.0 [0.0–2.9]	1.8 [0.3–5.3]	10.4% (− 28.7%, 71.2%)	0.66
AF episodes (min), median [25th–75th percentiles]	119.0 [2.3–617.0]	34.5 [0.0–2643.5]	78.0 [0.0–297.0]	53.7 [0.0–505.1]	84.7 [18.5–574.9]	− 28.9% (− 67.2%, 54.1%)	0.39
Maximal ventricular rate response (beats/minute), mean ± SD	124.8 ± 20.9	116.3 ± 32.7	106.7 ± 23.4	141.6 ± 30.6	143.4 ± 42.3	9.4 (1.2, 17.6) beats/minute	0.03

All outcomes, including the cumulative duration of AF and the number of AF episodes were reported for 3-month time windows

*AF* atrial fibrillation, *CI* confidence interval, *SD* standard deviation

^a^All observations (20 patients with 98 observations) were included. For observations of permanent AF the duration was imputed as 24 h (equal to 1440 min) of AF per day and 2190 h of AF per 3 months

^b^A subset of 16/20 patients with 78/98 observations was analyzed. All observations for patients experiencing any permanent AF ICM recordings were excluded

Based on 24-h Holter monitoring, heart rate remained stable (− 0.3 beats/min/year; 95% CI − 1.7, + 1.1; *p* = 0.68), while the number of ventricular ectopic beats increased over time (+ 54.8%/year; 95% CI + 19.7%, + 100.3%; *p* < 0.001) (Table 3). No changes were observed in the number of DDDs of antiarrhythmic drugs (− 12.5%, 95% CI − 28.4%, + 6.9%; *p* = 0.19) (Supplemental Table 1).

**Table 3:** Twenty-four hour Holter monitoring

	Baseline	6 months	1 year	2 years	3 years	Annual change (95% CI)	*p* value
	(*N* = 20)	(*N* = 19)	(*N* = 19)	(*N* = 19)	(*N* = 19)		
Heart rate (beats/minute), median [25th–75th percentiles]	71 [60–75]	66 [63–70]	71 [62–76]	68 [60–73]	68 [65–73]	− 0.3 (− 1.7, 1.1) beats/minute	0.68
Supraventricular ectopic beats (*n*), median [25th–75th percentiles]	187 [87–898]	218 [58–1173]	79 [26–564]	283 [79–608]	282 [99–1359]	15.2% (− 9.0%, 45.8%)	0.24
Ventricular ectopic beats (*n*), median [25th–75th percentiles]	35 [4–149]	22 [4–85]	42 [6–105]	29 [11–390]	89 [41–269]	54.8% (19.7%, 100.3%)	< 0.001

*CI* confidence interval, *SD* standard deviation

**Fig. 2:**
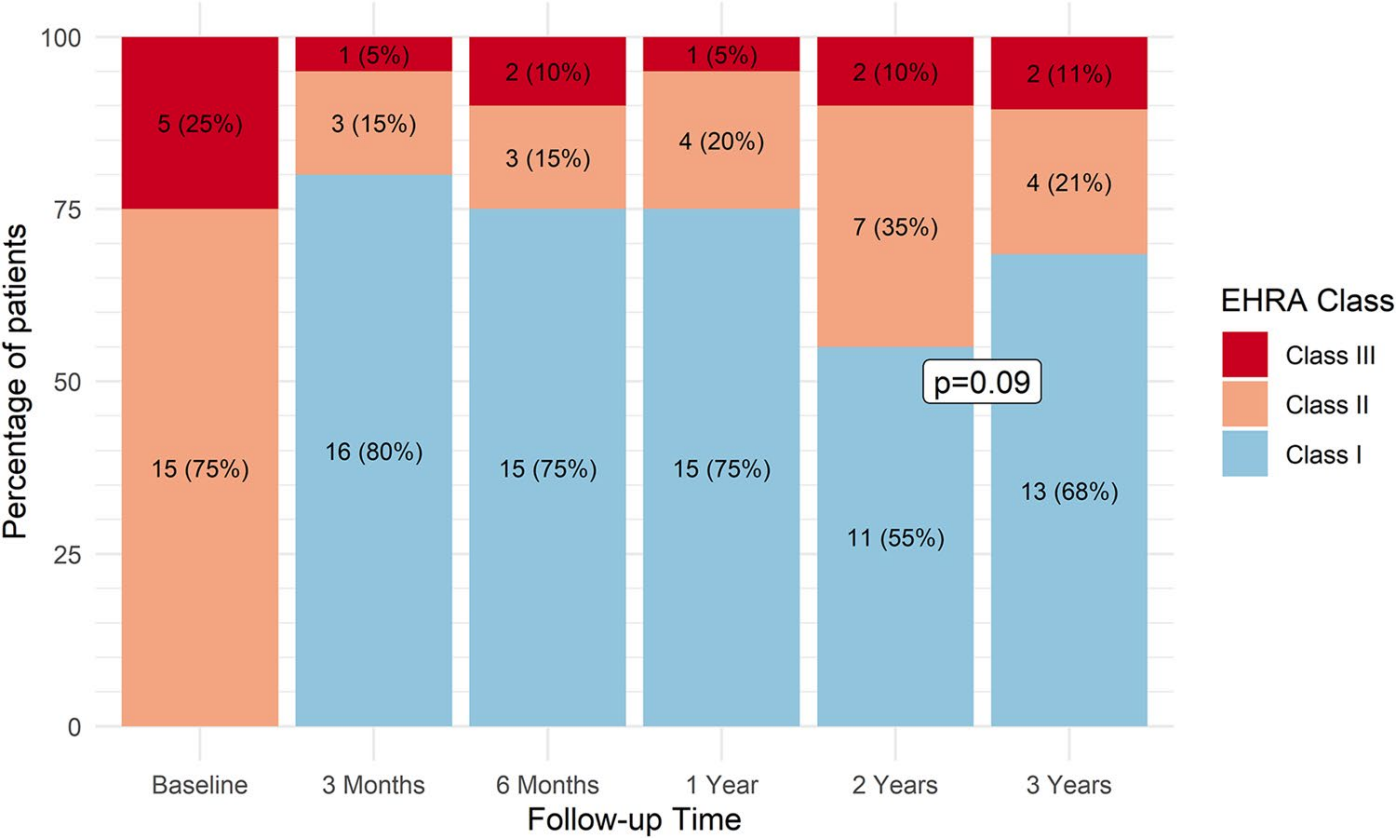
European Heart Rhythm Association (EHRA) symptomatology class over time

**Fig. 3:**
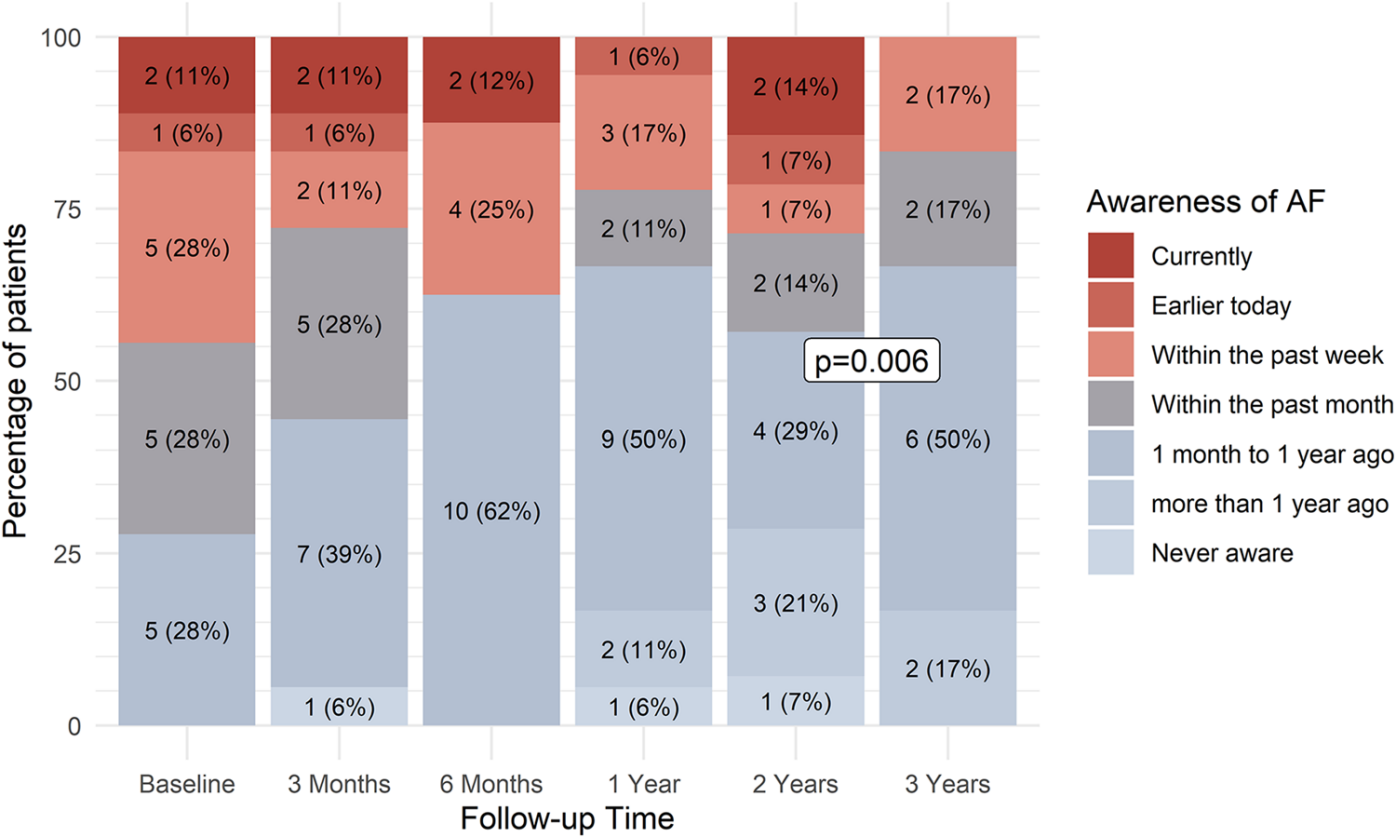
Patient-reported most recent awareness of atrial fibrillation as measured using the Atrial Fibrillation Effect on QualiTy-of-Life questionnaire

With respect to AF-related symptoms, the number of patients in EHRA class I numerically increased from 0 (0%) to 16 (80%) at 3 months and remained stable thereafter, thereby not reaching statistical significance (*p* = 0.09) (Fig. 2). The overall AFEQT score did not change over time (+ 1.6 points/year; 95% CI − 1.6, + 4.8; *p* = 0.32). However, patient-reported most recent awareness of AF significantly improved over time (*p* = 0.006) (Fig. 3).

Throughout follow-up, significant reductions in mean 24-h ambulatory systolic BP (− 2.2 mmHg/year; 95% CI − 3.9, − 0.6; *p* = 0.01) and diastolic BP (− 1.9 mmHg/year; 95% CI − 2.9, − 0.9; *p* < 0.001) were observed. In parallel, office systolic BP decreased (− 4.3 mmHg/year; 95% CI − 6.8, − 1.8; *p* = 0.001), while office diastolic BP remained stable (− 1.0 mmHg/year; 95% CI − 2.5, + 0.6; *p* = 0.21). No changes were observed with respect to the number of DDDs of antihypertensive drugs (− 3.5%/year; 95% CI − 13.3%, + 7.6%; *p* = 0.52) (Table 4).

**Table 4:** Ambulatory and office blood pressure and antihypertensive drug burden over time

Ambulatory blood pressure	Baseline	3 months	6 months	1 year	2 years	3 years	Annual change (95% CI)	*p *value
	(*N* = 20)	(*N* = 14)	(*N* = 17)	(*N* = 17)	(*N* = 17)	(*N* = 17)		
Mean 24-h SBP (mmHg), mean ± SD	129.5 ± 15.5	120.4 ± 9.8	120.5 ± 8.9	123.6 ± 10.9	121.2 ± 11.0	120.3 ± 12.7	− 2.2 (− 3.9, − 0.6) mmHg	0.01
Mean 24-h DBP (mmHg), mean ± SD	77.3 ± 9.3	73.2 ± 8.0	71.5 ± 6.2	74.3 ± 9.4	71.7 ± 6.5	69.9 ± 7.6	− 1.9 (− 2.9, − 0.9) mmHg	< 0.001
Daytime SBP (mmHg), mean ± SD	132.3 ± 15.8	124.0 ± 10.3	124.3 ± 8.4	126.6 ± 11.9	124.2 ± 11.2	122.8 ± 12.4	− 2.5 (− 4.2, − 0.8) mmHg	0.004
Daytime DBP (mmHg), mean ± SD	79.5 ± 9.7	76.1 ± 8.6	74.5 ± 5.5	76.6 ± 10.0	74.5 ± 6.5	72.2 ± 8.3	− 1.9 (− 3.0, − 0.9) mmHg	< 0.001
Nighttime SBP (mmHg), mean ± SD	120.7 ± 17.4	106.4 ± 12.1	110.4 ± 12.8	116.2 ± 11.8	113.4 ± 12.9	114.6 ± 16.7	− 0.6 (− 2.7, 1.5) mmHg	0.56
Nighttime DBP (mmHg), mean ± SD	70.1 ± 10.4	61.9 ± 6.0	63.9 ± 7.7	67.7 ± 10.2	64.9 ± 7.5	64.8 ± 7.8	− 0.8 (− 2.0, 0.4) mmHg	0.21

Office blood pressure	Baseline	3 months	6 months	1 year	2 years	3 years	Annual change (95% CI)	*p *value
	(*N* = 20)	(*N* = 20)	(*N* = 19)	(*N* = 20)	(*N* = 18)	(*N* = 13)		
Office SBP (mmHg), mean ± SD	153.8 ± 15.2	133.7 ± 15.6	146.5 ± 15.1	132.8 ± 17.0	132.6 ± 15.7	136.3 ± 11.4	− 4.3 (− 6.8, − 1.8) mmHg	0.001
Office DBP (mmHg), mean ± SD	87.5 ± 10.4	81.1 ± 8.3	80.4 ± 10.5	80.5 ± 9.4	83.1 ± 10.0	80.1 ± 7.0	− 1.0 (− 2.5, 0.6) mmHg	0.21

Antihypertensive drug summary measures^a^	Baseline	3 months	6 months	1 year	2 years	3 years	Annual change (95% CI)	*p *value
	(*N* = 20)	(*N* = 20)	(*N* = 20)	(*N* = 20)	(*N* = 20)	(*N* = 19)		
Number of DDDs, mean ± SD	2.7 ± 1.6	2.5 ± 1.4	2.7 ± 1.5	2.7 ± 1.6	2.5 ± 1.2	2.4 ± 1.6	− 3.5% (− 13.3%, 7.6%)	0.52
Number of antihypertensive drugs, median [25th–75th percentiles]	2.0 [2.0–3.0]	2.0 [1.8–3.0]	2.0 [2.0–3.0]	2.0 [1.8–3.0]	2.0 [1.8–2.3]	2.0 [1.0–3.0]	− 1.9% (− 12.8%, 10.5%)	0.76

*DBP * diastolic blood pressure, *DDD* defined daily dosage, *SBP* systolic blood pressure, *SD* standard deviation

^a^Betablockers were excluded from the antihypertensive drug burden calculation as they were included in the antiarrhythmic drug burden

### Safety outcomes

**Fig. 4:**
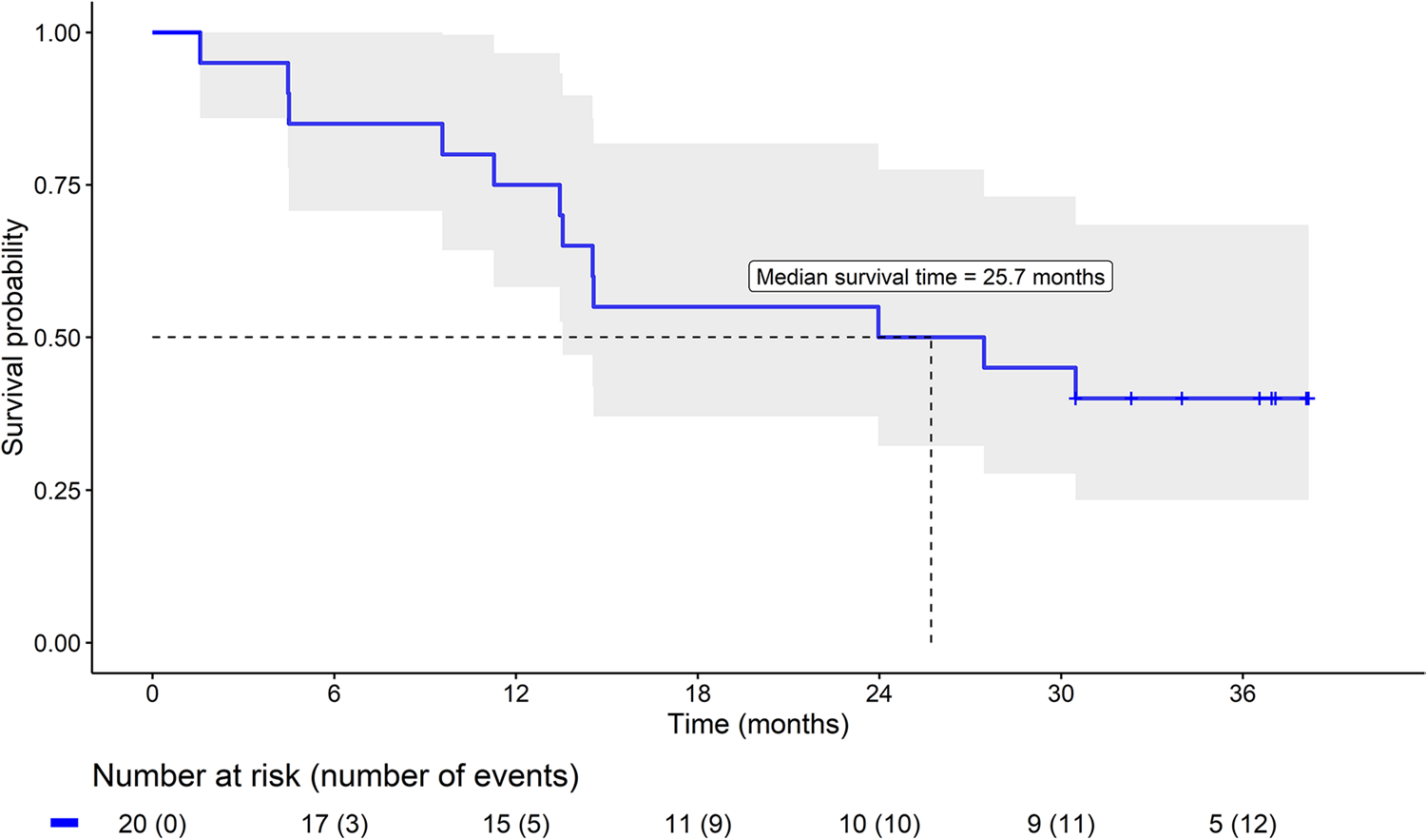
Arrhythmia-related event-free survival

During 3 years of follow-up, the primary safety outcome occurred in 12 (60%) patients while the median time-to-event was 25.7 [12.9–34.6] months (Fig. 4). The most frequently observed secondary safety outcomes were uptitration of antiarrhythmic drugs in 9 (47.5%) patients and electrical cardioversion in 7 patients (35.0%) (Table 5).

**Table 5:** Arrhythmia-related clinical events

	Cumulative incidence—% (number of events)					Median time-to-event—months [interquartile range]
	3 months	6 months	1 year	2 years	3 years	
Composite endpoint	5.0 (1)	15.0 (3)	25.0 (5)	50.0 (10)	60.0 (12)	25.7 [12.9–34.6]
Individual components						
Electrical cardioversion	5.0 (1)	10.0 (2)	20.0 (4)	35.0 (7)	35.0 (7)	NA
Chemical cardioversion	0.0 (0)	0.0 (0)	0.0 (0)	0.0 (0)	0.0 (0)	NA
Pulmonary vein isolation	0.0 (0)	0.0 (0)	0.0 (0)	5.0 (1)	10.3 (2)	NA
MAZE procedure	0.0 (0)	0.0 (0)	0.0 (0)	5.0 (1)	16.7 (3)	NA
First uptitration of antiarrhythmic drugs as compared to baseline	0.0 (0)	5.0 (1)	5.0 (1)	35.0 (7)	47.5 (9)	NA
First increase in EHRA class as compared to baseline	0.0 (0)	0.0 (0)	5.0 (1)	5.0 (1)	18.7 (3)	NA

## Discussion

To the best of our knowledge, this pilot study was the first to report on the long-term safety and efficacy of RDN as a stand-alone treatment modality for hypertensive patients with symptomatic AF. Throughout a 3-year follow-up period, no sustained significant reduction in AF burden was observed following radiofrequency RDN.

Within the current study, we observed a numerical decline in AF burden within the first 6 months following RDN, which was followed by a stabilization out to 3 years. Overall, no significant change in AF burden was observed following RDN. AF burden at baseline and throughout follow-up was assessed using ICM interrogation as this technique was previously identified as an accurate method for quantification of arrhythmia burden in AF patients undergoing interventional therapies [20]. Previous data demonstrated sensitivity and specificity figures of 100% and 86% for the detection of AF for the particular ICM used in the current study [21].

One of the most intriguing findings of our study was the low burden of AF as measured in minutes/day using ICM interrogation. Despite having symptoms significant enough to consider device-based treatment options for AF, the median burden of AF was merely 1.4 min/day. The latter confirms recent findings from other studies in the field, that demonstrated similarly low AF burden rates using ICM interrogation, ranging between 0.13% (1.9 min/day) and 3.60% (51.8 min/day) [22–25]. Of interest, in asymptomatic patients without a history of supraventricular arrhythmias, AF was still diagnosed in 28–35% when using an ICM and AF burden varied between 0.07% (1.0 min/day) and 0.12% (1.7 min/day) [26, 27]. Overall, the variety in quantified AF burden between several clinical studies in symptomatic and asymptomatic patients emphasizes the weak correlation between the severity of AF-related symptoms and the actual AF burden as measured using an ICM. This finding was accentuated by our data, which displayed high frequencies of EHRA II and III class symptoms (75% and 25%, respectively) while only a moderate AF burden was captured using ICM interrogation. The low AF burden in the current study may have influenced our results, as any proportionally substantial reduction in AF burden would be hardly detectable due to the small absolute magnitude of effect.

This study on stand-alone RDN treatment in hypertensive patients with symptomatic AF should be seen in the perspective of previous studies that focused on RDN as a concomitant procedure during other invasive antiarrhythmic procedures such as PVI [14]. RDN in addition to PVI demonstrated a 38% reduction in AF recurrence rate as compared to PVI alone in a recent meta-analysis [14]. However, none of the trials incorporated used ICMs to monitor AF burden. Furthermore, a substantial number of small sample size studies were included and publication bias cannot be ruled out.

Throughout 3 years of follow-up, we observed a sustained increase in the maximal VRR during episodes of AF as well as in the number of ventricular ectopic beats (during 24-h Holter monitoring). While these numbers cannot be readily explained, these findings could potentially be related to progression in AF severity over time, to a change in classes of prescribed antiarrhythmic drugs or a change in patient adherence to antiarrhythmic medication.

With respect to treatment safety, a total of 12 patients (60%) met the definition of the primary composite safety endpoint. This number was mainly driven by the number of patients that required electrical cardioversion or intensification of antiarrhythmic therapy. None of the adverse events observed were considered related to the RDN procedure, which supports the low complication rates displayed in the literature [7–13].

Furthermore, our findings on BP reduction were in line with those of previous randomized sham-controlled trials which demonstrated a significant reduction in BP or antihypertensive drug burden following RDN [7–13]. Of interest, patients with a history of AF were excluded from several RDN trials, thereby limiting the generalizability of their conclusions to the population currently studied [7–9, 12]. Within the current pilot study, we observed a sustained decrease in mean 24-h ambulatory SBP (− 2.2 mmHg/year) and office SBP (− 4.3 mmHg/year) out to 3 years. These findings support a durable BP-lowering effect of RDN also in patients with paroxysmal or persistent AF. In large population studies, antihypertensive drug treatment demonstrated to lower cardiovascular risk also in patients with AF [28, 29]. It remains unknown whether this effect is mediated entirely through the beneficial vascular effects of BP reduction, or also through a secondary pathway related to the protective effect of a decrease in AF burden. Given our current findings post RDN, involving a significant BP decrease while AF burden remains stable, it would be interesting to learn more about this physiological mechanism. Dedicated prospective randomized trials are needed to provide more insights into the relation between BP, AF burden and cardiovascular risk in AF patients.

### Future study recommendations

Given the small sample size of virtually all previous studies on the topic, their results should be interpreted with caution, especially in relation to the substantial risk of biases similar to those previously observed in RDN trials in hypertensive patients. It seems imperative that future prospective studies investigating the efficacy of RDN in the treatment of AF should be carefully designed, including complete blinding using a sham-control group while closely monitoring changes in prescribed drug regimen and patient adherence throughout follow-up [30]. Lastly, given the poor correlation between signs and symptoms of AF, arrhythmia burden should be quantified using standardized, continuous measurement of AF burden (such as ICM interrogation) rather than using clinical arrhythmia recurrence data.

### Limitations

First, this pilot study was not statistically powered to detect a predefined difference in any of the study outcomes. Therefore, we cannot rule out that our negative findings were caused by a lack of statistical power. Second, the absence of a sham-control arm precludes any causal statements on RDN and the change in outcome measures over time. Third, the RDN device used to treat patients in this study is not commercially available anymore, due to discontinuation of the product pipeline, not related to specific concerns about the efficacy or the safety of the device. Therefore, the results of novel studies investigating the safety and efficacy of currently commercially available ultrasound, radiofrequency or ethanol-based RDN devices in patients with AF are eagerly awaited. With regard to renal artery anatomy, the current study focused on main renal artery branch RDN using the EnligHTN™ system. Whether radiofrequency-based RDN using more contemporary technologies in both main and side branches would have resulted in different findings remains to be determined. Finally, no adherence measurements for antiarrhythmic or antihypertensive drug therapy were performed, thereby precluding any statements on the effect of changes in therapy adherence throughout the course of the study.

## Conclusion

In patients with a history of hypertension and symptomatic paroxysmal or permanent AF, stand-alone RDN resulted in a significant BP-lowering effect but did not affect AF burden up to 3 years of follow-up.

### Supplementary Information

Below is the link to the electronic supplementary material.

Supplementary file1 (PDF 314 KB)
