# Supplementary material for: Long-term safety and efficacy of renal sympathetic denervation in atrial fibrillation: 3-year results of the AFFORD study
Source: Clin Res Cardiol. 2023 May 25;112(12):1766–77. doi: 10.1007/s00392-023-02222-3 (PMC10697905; doi:10.1007/s00392-023-02222-3)
Supplement: Supplementary file 1 — Supplementary file1 (PDF 314 KB) [file 392_2023_2222_MOESM1_ESM.pdf]

Supplemental Material

Supplemental Table 1. Antiarrhythmic drug burden over time

	Baseline	6 months	1 year	2 years	3 years	Annual change (95% CI)	P-value
	(N=20)	(N=20)	(N=20)	(N=20)	(N=19)		
**Antiarrhythmic drug summary measures**							
Number of DDDs, median [25th-75th percentile]	1.0 [0.5-1.3]	1.0 [0.5-1.1]	0.8 [0.5-1.0]	0.9 [0.3-1.0]	0.5 [0.3-1.0]	-12.5% (-28.4%, 6.9%)	0.19
Number of antiarrhythmic drugs, median [25th-75th percentile]	1 [1.0-1.3]	1.0 [1.0-1.0]	1.0 [1.0-1.0]	1.0 [1.0-1.0]	1.0 [1.0-1.0]	-7.2% (-21.6%, 9.9%)	0.39
**Individual classes of antiarrhythmic drugs**							
Class I, n (%)	3 (15.0)	3 (15.0)	3 (15.0)	3 (15.0)	1 (5.3)		
Class II, n (%)	6 (30.0)	5 (25.0)	5 (25.0)	6 (30.0)	6 (31.6)		
Class III, n (%)	13 (65.0)	12 (60.0)	13 (65.0)	9 (45.0)	9 (47.4)		
Class IV, n (%)	0 (0.0)	0 (0.0)	0 (0.0)	0 (0.0)	0 (0.0)		
Glycosides, n (%)	2 (10.0)	2 (10.0)	2 (10.0)	2 (10.0)	2 (10.5)		

CI, Confidence Interval. DDD, Defined Daily Dosage.
